# Signaling through TLR5 mitigates lethal radiation damage by neutrophil-dependent release of MMP-9

**DOI:** 10.1038/s41420-021-00642-6

**Published:** 2021-09-28

**Authors:** Craig M. Brackett, Kellee F. Greene, Alyssa R. Aldrich, Nicholas H. Trageser, Srabani Pal, Ivan Molodtsov, Bojidar M. Kandar, Lyudmila G. Burdelya, Scott I. Abrams, Andrei V. Gudkov

**Affiliations:** 1 Department of Cell Stress Biology, Roswell Park Comprehensive Cancer Center, Buffalo, NY 14263 USA; 2 I.V. Davydovsky Clinical City Hospital, Moscow Department of Healthcare, Moscow, Russian Federation; 3 Department of Immunology, Roswell Park Comprehensive Cancer Center, Buffalo, NY 14263 USA

**Keywords:** Neutrophils, Regeneration, Acute inflammation

## Abstract

Acute radiation syndrome (ARS) is a major cause of lethality following radiation disasters. A TLR5 agonist, entolimod, is among the most powerful experimental radiation countermeasures and shows efficacy in rodents and non-human primates as a prophylactic (radioprotection) and treatment (radiomitigation) modality. While the prophylactic activity of entolimod has been connected to the suppression of radiation-induced apoptosis, the mechanism by which entolimod functions as a radiomitigator remains poorly understood. Uncovering this mechanism has significant and broad-reaching implications for the clinical development and improvement of TLR5 agonists for use as an effective radiation countermeasure in scenarios of mass casualty resulting from accidental exposure to ionizing radiation. Here, we demonstrate that in contrast to radioprotection, neutrophils are essential for the radiomitigative activity of entolimod in a mouse model of lethal ARS. Neutrophils express functional TLR5 and rapidly exit the bone marrow (BM), accumulate in solid tissues, and release MMP-9 following TLR5 stimulation which is accompanied by an increase in the number of active hematopoietic pluripotent precursors (HPPs) in the BM. Importantly, recombinant MMP-9 by itself has radiomitigative activity and, in the absence of neutrophils, accelerates the recovery of the hematopoietic system. Unveiling this novel TLR5-neutrophil-MMP-9 axis of radiomitigation opens new opportunities for the development of efficacious radiation countermeasures to treat ARS following accidental radiation disasters.

## Introduction

Exposure to ionizing radiation causes significant DNA damage in many cells of the organism and is the underlying cause of multiple pathologies including acute radiation sickness (ARS). They can occur either accidentally (i.e., nuclear disasters like Chernobyl of Fukushima catastrophes) or intentionally (i.e., cancer therapies) and require the development of countermeasures that can reduce the severity of ARS and improve recovery and survival. Such countermeasures are classified as either protectants when given prophylactically prior to intentional radiation or mitigators when administered therapeutically after radiation exposure [1].

The development of protectors has been more successful than radiomitigators. The only FDA-approved powerful radioprotective drug, an antioxidant amifostine, has no efficacy as a radiomitigator [2]. In fact, dramatic cell loss in the hematopoietic system (HPS) due to p53-mediated apoptosis induced by DNA damage occurs within the first few hours after radiation exposure [3, 4] and renders radioprotective agents completely ineffective. Similarly, agents that induce proinflammatory cytokines (e.g., TNF, IL-12) [5, 6] are another well-studied class of efficacious radioprotectants that commonly act as activators of a pro-survival p53-suppressive NF-κB pathway [7].

Efficacious radiomitigators engage mechanisms of tissue regeneration and reduce the risk of sepsis and bleeding rather than suppressing apoptosis [8]. The only class of FDA-approved drugs for such indications are derivatives of G-CSF (Neupogen and Neulasta) commonly used in oncology to accelerate the recovery of hematopoiesis following chemotherapy-associated myelosuppression [1]. Unfortunately, the efficacy of these G-CSF-based drugs is rather weak, and they require multiple administrations under conditions of supportive clinical care [9–11] and do not satisfy the needs of mass casualty scenarios. Thus, the development of potent yet feasible mitigating countermeasures remains a strong unmet medical need.

To that end, our prior work defined bacterial flagellin, the sole toll-like receptor (TLR) 5 agonist, as a superior radioprotectant when compared to amifostine for suppressing apoptosis in radiosensitive tissues [12]. We developed a pharmacologically optimized flagellin derivative, entolimod, as a powerful radioprotector in mice and non-human primates but, unlike amifostine, did not diminish the radiosensitivity of tumors [12]. Moreover, entolimod appeared to be an effective radiomitigator in rodent and non-human primate models of lethal ARS caused by total body irradiation (TBI). A single dose of entolimod with no supportive care rescued animals when administered within 48 h post-TBI [13, 14] thus exceeding the efficacy of G-CSF-based drugs. Remarkably, entolimod also showed efficacy in other pre-clinical models of both genotoxic [15–17] and non-genotoxic stressors [18–20].

While entolimod-induced radioprotection is well-known to involve a combination of anti-apoptotic, anti-oxidative, and anti-bacterial effects elicited by the liver [12, 19], the mechanism of radiomitigation remains poorly understood. Uncovering this mechanism could guide the development of more effective and safe countermeasures to treat patients with ARS. To determine the mechanism by which entolimod mitigates radiation damage, we focused on the cellular effectors of its function. In our prior studies of anticancer immunotherapeutic effects of entolimod, we observed a profound neutrophil (Nϕ) response as one of the earliest cellular consequences of systemic TLR5 activation characterized by release from the BM, rapid exit from the blood, and recruitment to tissues [19]. This Nϕ response was defined as one of three biomarkers of efficacy for entolimod, along with increased systemic levels of G-CSF and IL-6 [13, 14].

Since entolimod presumably mitigates radiation damage by improving HPS recovery and the release of matrix metalloproteinase-9 (MMP-9) by Nϕ has been linked to stimulating hematopoiesis [21], we hypothesized that entolimod mitigates radiation damage to the HPS through Nϕ-dependent release of MMP-9. Using a mouse model of lethal ARS induced by TBI to test this hypothesis, we found that Nϕ (i) are essential mediators of the radiomitigative but not radioprotective abilities of entolimod, (ii) express functional TLR5 but undergo minimal transcriptional changes post-entolimod suggesting that Nϕ mitigate ARS through a transcriptional-independent mechanism; and (iii) increase the number of active hematopoietic pluripotent precursors (HPPs) in bone marrow, which can be mimicked by administration of recombinant pro-MMP-9 (rMMP-9) in the absence of Nϕ. These results define the release of MMP-9 by Nϕ as a major contributor, along with G-CSF and IL-6 [14], as mediators of radiomitigation post-entolimod by increasing the number of active HPPs to facilitate recovery of a damaged HPS. Altogether, these data underscore the potential use of MMP-9 to compensate for the shortcomings of currently approved radiation countermeasures.

## Results

### Nϕ are essential mediators of the radiomitigative but not radioprotective abilities of entolimod

**Fig. 1:**
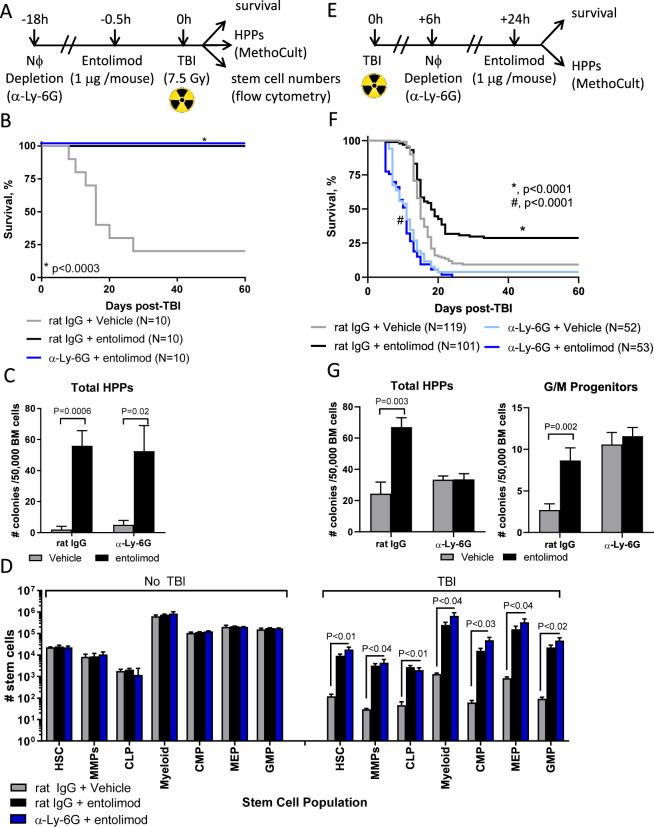
Nϕ mediate the radiomitigative but not radioprotective abilities of entolimod. Experimental layouts to determine the role of Nϕ in the **A** radioprotective and **E** radiomitigative effects of entolimod in BALB/c mice. Survival by Kaplan–Meier curves in mice treated with entolimod in the presence (rat IgG) or absence (α-Ly-6G) of Nϕ in the **B** radioprotective and **F** radiomitigative schemes. *P*-values were determined by Log-rank test. Measurement of total HPPs and granulocyte/macrophage (G/M) progenitors in BM by MethoCult in the **C** radioprotective (*n* = 4–5 mice /group) and **G** radiomitigative (*n* = 3–10 mice/group) schemes. Total HPPs were measured +1 h post-TBI for radioprotection and for radiomitigation, both total HPPs and G/M progenitors +3d post-treatment with vehicle or entolimod. **D** Absolute number of stem cell populations in BM by flow cytometry in the presence (rat IgG) and absence (α-Ly-6G) of Nϕ on day 8 post-TBI in the radioprotection scheme (*n* = 3–5 mice/group). Stem cell populations were defined as follows: HSC (Lineage^−^ Flt3^−^ c-kit^+^ Sca-1^+^), MMP (Lineage^−^ Flt3^+^ c-kit^+^ Sca-1^+^), CLP (Lineage^−^ Flt3^−^ IL-7R^+^ c-kit^+^ Sca-1^+^), Myeloid (Lineage^−^ Flt3^−^ IL-7R^−^ c-kit^−^ Sca-1^−^), CMP (Lineage^−^ Flt3^−^ IL-7R^−^ CD34^+^ CD16/32^−^), MEP (Lineage^−^ Flt3^−^ IL-7R^−^ CD34^−^ CD16/32^−^), and GMP (Lineage^−^ Flt3^−^ IL-7R^−^ CD34^+^ CD16/32^+^). Error bars represent mean ± SEM; *P*-values were determined by Student’s *t*-test.

Identification of Nϕ as one of three biomarkers of entolimod’s efficacy as a radiation countermeasure [13] prompted us to determine whether Nϕ are cellular mediators of radioprotection and radiomitigation. We accomplished this using a loss-of-function approach to deplete Nϕ in vivo using the well-accepted α-Ly6G antibody [22], which effectively depletes Nϕ by at least 95% (Supplementary Fig. S1), prior to entolimod administration in the radioprotective (Fig. 1A) and radiomitigative (Fig. 1E) schemes similarly used by us [12–16, 19].

In the radioprotection scheme, entolimod protected inbred BALB/c (Fig. 1B) and outbred NIH-S (Supplementary Fig. S2A) mice from lethal ARS both in the presence (rat IgG) and absence (α-Ly6G) of Nϕ. Consistently, Nϕ depletion did not diminish the ability of entolimod to protect the number of total HPPs as measured by MethoCult in both BALB/c (Fig. 1C) and NIH-S (Supplementary Fig. S2B) mice. Moreover, the number of stem cell populations in the BM of BALB/c (Fig. 1D) and NIH-S (Supplementary Fig. S2C) mice recovered in the presence and absence of Nϕ and was remarkably similar to stem cell numbers in control mice that did not receive TBI. Thus, the radioprotective ability of entolimod does not depend on Nϕ.

In stark contrast to radioprotection, Nϕ depletion in BALB/c mice abrogated the radiomitigative activity of entolimod (Fig. 1F). Intriguingly, Nϕ-depleted mice portrayed worse overall survival than mice with Nϕ suggesting a critical role for Nϕ in the global response of an organism to radiation damage. The absence of Nϕ also prevented the recovery of both total BM HPPs and granulocyte/macrophage (G/M) progenitors (Fig. 1G). Collectively, these data show that entolimod facilitates radioprotection and radiomitigation through distinct mechanisms, whereby, Nϕ are essential for the radiomitigative but not radioprotective activities of entolimod.

### Entolimod augments Nϕ recruitment and expression of homing molecules

**Fig. 2:**
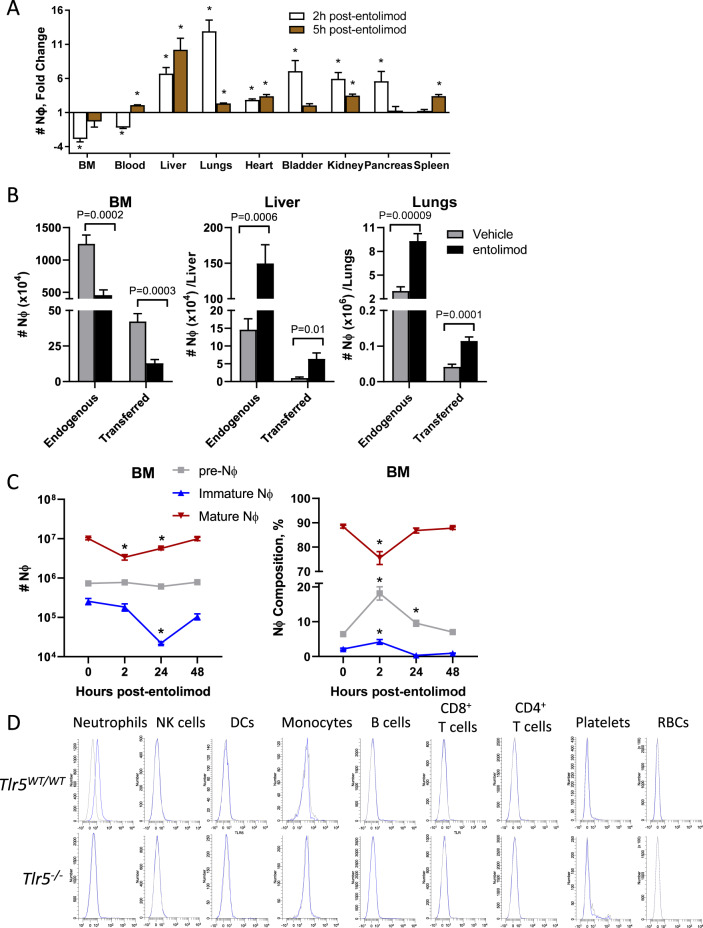
Entolimod stimulates Nϕ recruitment to tissues and differentiation. **A** Fold change in the Nϕ response in the indicated organs calculated from the absolute number of Nϕ in entolimod-treated (2 h and 5 h) versus vehicle-treated mice (*n* = 3–5 mice /group). Flow cytometry was used to identify Nϕ (CD45^+^ CD11b^+^ Ly-6C^+^ Ly-6G^hi^). **B** Absolute number of endogenous and adoptively transferred Nϕ in the BM, liver, and lungs 2 h post-treatment with vehicle or entolimod (*n* = 6–8 mice/group). Adoptive transfers were done by i.v. injection of GFP-expressing BM immediately before treatment. Endogenous versus transferred Nϕ were distinguished by flow cytometry for GFP among the total Nϕ population (CD45^+^ CD11b^+^ Ly-6C^+^ Ly-6G^hi^). **C** Absolute number (left) and composition (right) of Nϕ populations in the bone marrow at the indicated time points post-treatment by flow cytometry as defined by: pre-Nϕ (Lineage^−^ c-kit^int^ CD115^−^ CD11b^+^ Gr-1^+^ CXCR4^+^), immature Nϕ (Lineage^−^ c-kit^int^ CD115^−^ CD11b^+^ Gr-1^+^ CXCR4^−^ CXCR2^−^ Ly-6G^+^), and mature Nϕ (Lineage^−^ c-kit^int^ CD115^−^ CD11b^+^ Gr-1^+^ CXCR4^−^ CXCR2^+^ Ly-6G^+^) where lineage was defined as CD3^−^ and B220^−^ (*n* = 3–9 mice/group). **D** Flow cytometry-based staining for TLR5 using biotinylated-entolimod plus streptavidin fluorochrome “sandwich” platform on Nϕ (CD45^+^ CD11b^+^ Ly-6C^+^ Ly-6G^hi^), NK cells (CD45^+^ CD3ε^−^ NKp46^+^), DCs (CD45^+^ Ly-6G^−^ B220^−^ F4/80^−^ CD11c^+^), monocytes (CD45^+^ Ly-6G^−^ B220^−^ CD11c^−^ F4/80^+^), B cells (CD45^+^ Ly-6G^−^ CD11c^−^ F4/80^−^ B220^+^), CD8^+^ T cells (CD45^+^ CD3ε^+^ CD8^+^), CD4^+^ T cells (CD45^+^ CD3ε^+^ CD4^+^), platelets (CD45^−^ CD41^+^), and RBCs (CD45^−^ Ter119^+^) in the blood of BALB/c and *Tlr5*^*−/−*^ mice. Error bars represent mean ± SEM. *P < 0.04; *P*-values were determined by Student’s *t*-test.

We next sought to perform a more comprehensive analysis of Nϕ recruitment to tissues post-entolimod, since our prior work only evaluated the release of Nϕ from the BM and recruitment to the liver [19]. At 2 h post-entolimod, Nϕ exited both the BM and the blood and were also recruited to all tissues examined except for the spleen (Fig. 2A). At 5 h post-entolimod, increased Nϕ recruitment was still observed in many tissues examined (including the liver, lungs, heart, and kidneys) in addition to the blood and spleen. Adoptively transferred Nϕ were also similarly released from the BM and recruited to the liver and lungs, two of the most prominent sites of recruitment post-entolimod treatment (Fig. 2B). These results indicate that entolimod stimulates Nϕ release from the BM and recruitment to tissues through a blood-borne homing mechanism.

Since Nϕ differentiation occurs across a linear path from pre-Nϕ and then to immature and mature Nϕ [23], we sought to understand whether entolimod stimulates the release of certain Nϕ populations from the BM. Entolimod only causes a drop in the number of mature Nϕ (Fig. 2C, left), indicating a preferential release of this Nϕ population. Entolimod also changes the composition of the Nϕ population in the BM as observed by a decrease in mature Nϕ and an increase in the percentages of both pre- and immature Nϕ (Fig. 2C, right). Thus, entolimod stimulates the release of mature Nϕ from the BM which, in turn, triggers differentiation of both pre-Nϕ and immature Nϕ.

Because reliable commercially available antibodies to detect TLR5 do not exist, we developed a flow cytometry-based “sandwich” platform that utilizes biotinylated-entolimod (b-entolimod) and a streptavidin-conjugated fluorochrome in combination with antibodies to identify which immune cell subsets express TLR5. Using this approach, and for additional specificity, we compared TLR5 expression in *Tlr5*^*WT/WT*^ vs. *Tlr5*^*−/−*^ mice. We found that Nϕ from the blood of wild-type mice are the only immune cells that bound b-entolimod (Fig. 2D). Importantly, Nϕ from *Tlr5*^*−/−*^ mice did not display staining, demonstrating that this approach specifically detects TLR5 expression. These results are consistent with prior observations showing that Nϕ express TLR5 [24].

We lastly sought to determine whether TLR5 signaling through MyD88, the canonical adapter for TLR signaling [25], downregulates L-selectin and upregulates CD11b on Nϕ in order to support adhesion to the endothelium and extravasation from the bloodstream into tissues [26–29]. Indeed, L-selectin was downregulated and CD11b was upregulated on Nϕ as early as 10 min post-entolimod and lasted for at least 120 min (Supplementary Fig. S3A). Near-complete abrogation of L-selectin downregulation and CD11b upregulation post-entolimod was observed in *Myd88*^*−/−*^ Nϕ, which was not due to a loss of cell-surface expression of TLR5 on *Myd88*^*−/−*^ Nϕ (Supplementary Fig. S3B) and is consistent with prior observations using a TLR4 agonist [30]. Entolimod-treated Nϕ had improved binding to endothelial cells and was significantly higher when both Nϕ and endothelial cells were pretreated with entolimod (Supplementary Fig. S3C). Lastly, blocking either L-selectin or ICAM-1, the ligand for CD11b, with antibodies, significantly attenuated Nϕ recruitment to the liver (Supplementary Fig. S3D). Taken together, these data demonstrate that entolimod stimulates Nϕ recruitment to tissues by triggering MyD88-dependent L-selectin downregulation and CD11b upregulation.

### Activation of TLR5 on Nϕ and non-hematopoietic cells cooperate to mitigate radiation damage

**Fig. 3:**
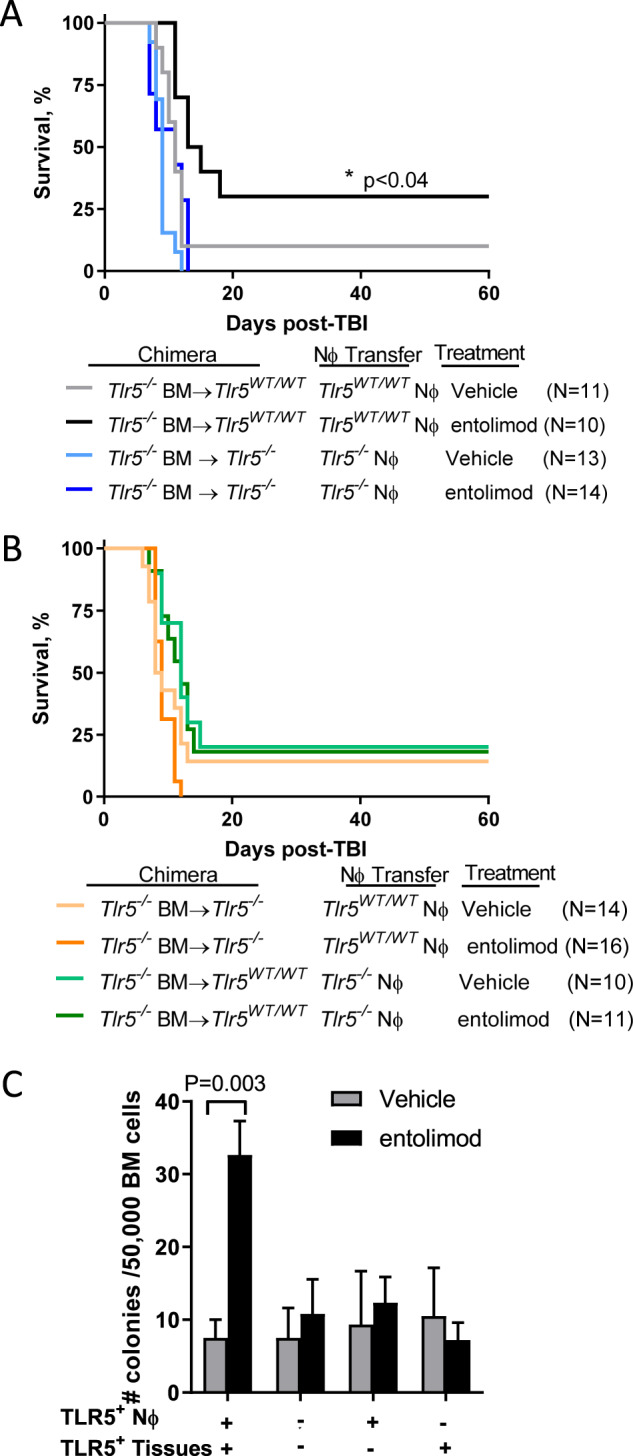
TLR5 activation on Nϕ and non-hematopoietic cells cooperate to mitigate radiation damage. *Tlr5*^*−/−*^ bone marrow chimeric mice received an adoptive transfer of either *Tlr5*^*WT/WT*^ or *Tlr5*^*−/−*^ Nϕ (10 million /mouse) followed immediately by vehicle or entolimod 24 h post-TBI. **A**, **B** Survival was measured by Kaplan–Meier curves. *P*-values were determined by Log-rank test; *n* = 10–16 mice/group. **C** Measurement of total HPPs by MethoCult in BM on day 7 post-treatment using the same experimental setup as **A**. Error bars represent mean ± SEM; *P*-values were determined by Student’s *t*-test; *n* = 4–7 mice/group. In all instances, Nϕ purity was routinely >98%.

Expression of functional TLR5 on both Nϕ (Fig. 2D) and non-hematopoietic cells (i.e., hepatocytes) [19] prompted us to determine the relative contribution of both cell populations to the radiomitigative activities of entolimod. To do this, we performed *Tlr5*^*WT/WT*^ (wild-type) or *Tlr5*^*−/−*^ Nϕ transfers in *Tlr5*^*−/−*^ bone marrow chimeras so that (i) both cell populations expressed TLR5 (wild-type Nϕ into *Tlr5*^*−/−*^ BM → wild-type chimeras); (ii) neither cell population expressed TLR5 (*Tlr5*^*−/−*^ Nϕ into *Tlr5*^*−/−*^ BM → *Tlr5*^*−/−*^ chimeras); (iii) only Nϕ expressed TLR5 (wild-type Nϕ into *Tlr5*^*−/−*^ BM → *Tlr5*^*−/−*^ chimeras); and (iv) only non-hematopoietic cells expressed TLR5 (*Tlr5*^*−/−*^ Nϕ into *Tlr5*^*−/−*^ BM → wild-type chimeras). Entolimod significantly improved overall survival when both cell populations expressed TLR5 (black vs. gray curves, Fig. 3A). The radiomitigative activity of entolimod was abrogated when both populations were TLR5-deficient (dark blue vs. light blue curves, Fig. 3A), which is further substantiated by the inability of entolimod to improve survival and stimulate HPPs recovery in the BM of *Tlr5*^*−/−*^ mice post-TBI (Supplementary Fig. S4). Entolimod was unable to mitigate radiation damage when either Nϕ (dark orange vs. light orange) or non-hematopoietic cells (dark green vs. light green) expressed TLR5 (Fig. 3B). Consistent with these findings, accelerated recovery of HPPs in the BM was only observed when both Nϕ and non-hematopoietic cells express TLR5 (Fig. 3C). Thus, these data show that stimulating TLR5 on both Nϕ and non-hematopoietic cells (i.e., hepatocytes) is required for mitigating lethal ARS.

### Release of MMP-9 by entolimod-stimulated Nϕ mitigates lethal radiation damage

Our prior work showing that G-CSF and IL-6 contribute to the radiomitigative activity of entolimod [14] led us to determine whether Nϕ mitigate ARS post-entolimod treatment by mediating the release of these cytokines. Depletion of Nϕ with α-Ly6G prior to entolimod treatment did not diminish the expression of either G-CSF or IL-6 in serum (Supplementary Fig. S5A). Moreover, entolimod-stimulated comparable serum levels of both cytokines in bone marrow chimeric mice expressing TLR5 in either non-hematopoietic cells or both Nϕ and non-hematopoietic cells (Supplementary Fig. S5B). Thus, the critical impact of Nϕ in the radiomitigative activities of entolimod is not mediated by the release of G-CSF and IL-6.

Given that Nϕ express functional TLR5 and that the underlying effects of TLR5 agonists have been associated with NF-κB-dependent transcriptional events [19], we hypothesized that changes in the transcriptional profile of Nϕ post-entolimod mitigate ARS. However, RNA sequencing data analysis showed that ex vivo stimulation of Nϕ with entolimod caused changes in transcriptional profiles that were substantially weaker—both in the numbers of responsive genes and scales of changes—as compared with that of liver cells following in vivo treatment with entolimod (Supplementary Fig. S6). This observation suggests that mitigation of lethal ARS by Nϕ post-entolimod involves a transcriptional-independent mechanism.

**Fig. 4:**
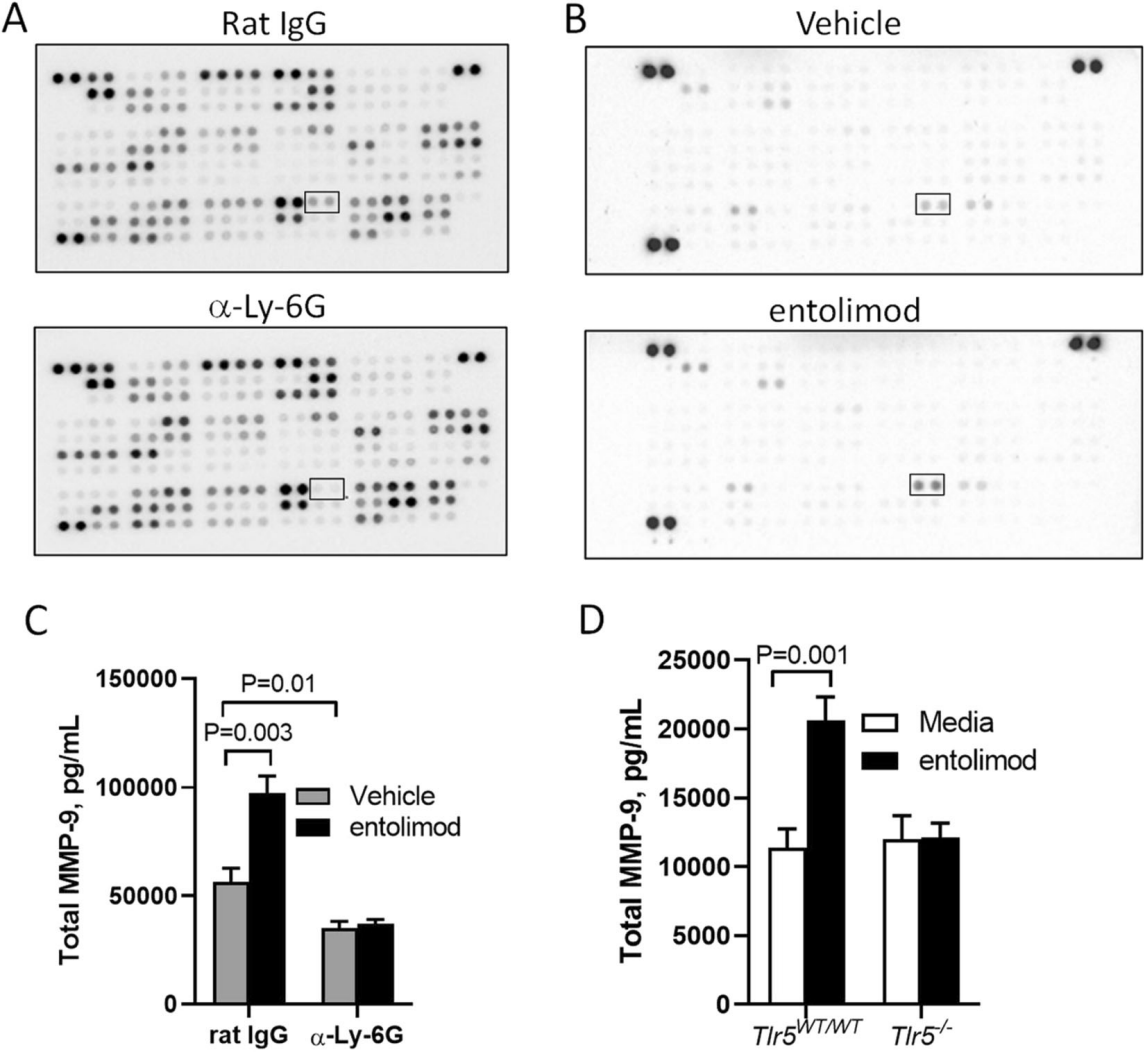
Entolimod stimulates Nϕ to release MMP-9. Protein microarray analysis of **A** mouse serum using R&D Systems Proteome Profiler Mouse XL Cytokine Array in the presence (rat IgG) and absence (α-Ly-6G) of Nϕ 24 h post-TBI and **B** Nϕ isolated from BM that were ex vivo treated with vehicle or entolimod for 30 min. Measurement by ELISA of MMP-9 in **C** serum from mice (*n* = 5/group) in the presence (rat IgG) and absence (α-Ly-6G) of Nϕ 2 h post-treatment with vehicle or entolimod (TBI was delivered 24 h prior to vehicle or entolimod) and **D** supernatants from *Tlr5*^*WT/WT*^ and *Tlr5*^*−/−*^ Nϕ isolated from BM (*n* = 6 mice/group) and ex vivo treated with vehicle or entolimod for 30 min.

To determine potential factors released by entolimod-stimulated Nϕ that contribute to the mitigation of ARS, we analyzed serum from mice that retained (rat IgG) or lacked (α-Ly6G) Nϕ using a protein microarray that simultaneously measures 111 soluble factors. We identified MMP-9 as the single factor reduced in the serum of Nϕ-depleted mice when compared to control-treated serum (Fig. 4A). Consistent with this finding, MMP-9 was also the only factor released by Nϕ into supernatants after ex vivo stimulation with entolimod (Fig. 4B). In support of the protein microarray data, Nϕ depletion prior to entolimod treatment abrogated the increased levels of MMP-9 in serum as measured by ELISA (Fig. 4C). Moreover, Nϕ released MMP-9 in supernatants following ex vivo stimulation in a TLR5-dependent manner (Fig. 4D). These data show that entolimod stimulates Nϕ to release MMP-9 and are consistent with prior observations that Nϕ are the main cellular source of MMP-9 [31–35].

**Fig. 5:**
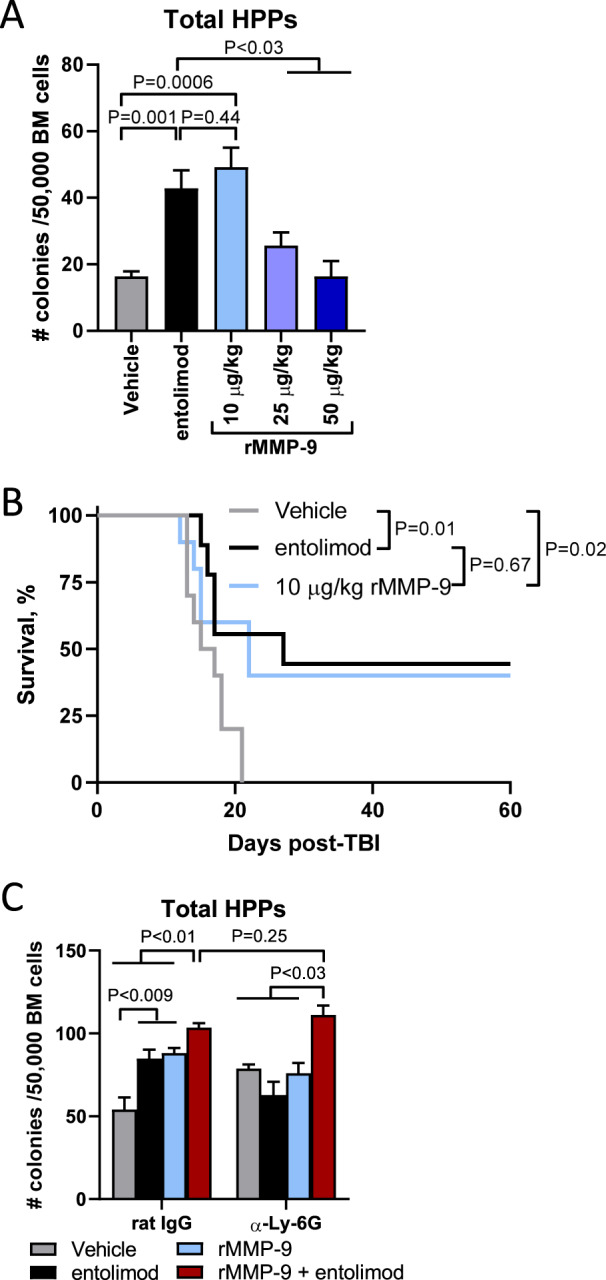
Release of MMP-9 by Nϕ post-entolimod mitigates lethal radiation damage to hematopoiesis. **A** BALB/c mice (*n* = 5/group) were given 5 Gy TBI followed 24 h later by treatment with vehicle, entolimod, or the indicated doses of recombinant pro-MMP-9 (rMMP-9). Measurement of total HPPs in BM by MethoCult was done on day 3 post-treatment. **B** Survival by Kaplan–Meier curve in BALB/c mice treated with vehicle, entolimod, or 10 μg/kg rMMP-9 24 h post 7.5 Gy TBI (*n* = 9–10 mice /group). *P*-values were determined by the Log-rank test. **C** BALB/c mice (*n* = 5/group) were given 5 Gy TBI followed 6 h later by rat IgG or α-Ly-6G. Twenty-four hours later mice were treated with vehicle, entolimod, rMMP-9 (10 μg/kg), or rMMP-9 plus entolimod. Measurement of total HPPs in BM by MethoCult was done on day 3 post-treatment. For **A** and **C** error bars represent mean ± SEM; *P*-values were determined by Student’s *t*-test.

Because the release of MMP-9 by Nϕ supports hematopoiesis [36, 37], we hypothesized that entolimod mitigates ARS through Nϕ-dependent release of MMP-9. We first measured whether the radiomitigative effects of rMMP-9 are linked to HPPs recovery in BM as measured by MethoCult. In fact, rMMP-9 administration 24 h post-TBI accelerated the recovery of HPPs in a dose-dependent manner with 10 μg/kg rMMP-9 showing optimal recovery similarly to entolimod (Fig. 5A). Moreover, significantly improved survival was observed following rMMP-9 administration and was strikingly similar to improved survival observed with entolimod (Fig. 5B). These data underscore that both entolimod and rMMP-9 mitigate lethal ARS.

Lastly, we sought to causally link the release of MMP-9 by entolimod-stimulated Nϕ to mitigation of ARS. To do this, mice that retained (rat IgG) or lacked (α-Ly6G) Nϕ were stratified into four treatment cohorts consisting of vehicle, entolimod, rMMP-9, and rMMP-9 plus entolimod (Fig. 5C). In the presence of Nϕ, both entolimod and rMMP-9 increased the number of BM HPPs with a modest but significant increase when both rMMP-9 and entolimod were administered. In Nϕ-deficient mice, HPPs only recovered to similar levels observed in Nϕ sufficient mice when both rMMP-9 and entolimod were administered. Collectively, these data show that both the release of MMP-9 by Nϕ and the inflammatory response (e.g., G-CSF) induced by TLR5 stimulation mitigate ARS.

## Discussion

The remarkable efficacy of TLR5 agonists as a radiomitigator has been recognized for many years, but a lack of mechanistic knowledge regarding which components of this multifunctional pathway rescue lethally irradiated organisms has significantly impeded the clinical development of TLR5 agonists as a radiation countermeasure following accidental exposure to ionizing radiation. Uncovering this mechanism is especially important provided the safety concerns of using bacterial protein (flagellin) derivatives, which are known to induce an IL-6-driven inflammatory response. Here, we provide evidence supporting the key role for the release of MMP-9 by TLR5-stimulated Nϕ as an essential mediator of radiomitigation.

We began this study by uncovering key differences in how the TLR5 agonist entolimod protects and mitigates radiation damage, which is reflected by the state of radiosensitive organs such as the HPS when a TLR5 agonist is administered. HPPs are intact prior to radiation thereby conferring protection from death by a series of pro-survival effects initiated by TLR5-mediated activation of NF-κB response including suppression of apoptosis (e.g., Bcl-2) and induction of natural endogenous antioxidants (e.g., superoxide dismutase) and anti-microbial factors (e.g., hepcidin) [12, 13, 19]. In stark contrast, post-irradiation treatment with radiomitigators requires rescuing an already damaged HPS that undergoes apoptotic death within the first 8 h post-TBI [38].

The previously identified mechanisms underlying the effects of TLR5 agonists have been associated with NF-κB-dependent transcriptional events [19] and prompted us to understand the transcriptome changes that occur in Nϕ post-entolimod. Although neutrophils express TLR5, we found minimal transcriptional changes in similar pathways that are markedly increased in the liver post-entolimod. Rather, Nϕ respond to entolimod by rapid and pronounced changes in proteins at the plasma membrane (L-selectin downregulation and CD11b upregulation) to support extravasation across endothelial walls and accumulation in tissues as is observed with other cell-surface TLRs [26–29]. Moreover, the release of MMP-9 by Nϕ is among the most striking microenvironment-changing factor post-TLR5 stimulation, which is post-transcriptionally released from tertiary granules following stimulation with a variety of mediators [31–35]. Since the release of MMP-9 by Nϕ is linked to stimulating hematopoiesis [21], we focused on MMP-9 as a potential contributor to Nϕ-mediated radiomitigative effects of TLR5 agonists.

MMP-9 is a metalloproteinase that is well-recognized for promoting tumor invasion and metastasis and thus is studied as a therapeutic target in cancer [reviewed in [39]]. Even though a series of reports demonstrated the involvement of MMP-9 in “opening” HP niches in order to promote hematopoietic recovery [36, 40], to the best of our knowledge considering recombinant MMP-9 (rMMP-9) as a pharmacologically feasible stimulator of blood regeneration following induction of systemic genotoxic stress (SGS) by radiation exposure has not been considered. Our data show that administering rMMP-9 has radiomitigative capabilities to strikingly similar levels as entolimod in terms of survival and accelerating the recovery of the number of BM HPPs. Moreover, rMMP-9 plus entolimod administration restores the radiomitigative capabilities of entolimod in the absence of Nϕ, demonstrating a causal link between TLR5 agonist-stimulated Nϕ and MMP-9 as a radiomitigator. These findings are supported by the known ability of MMP-9 to cleave several cytokines and/or their receptors, a process that can activate cytokines playing a role in cellular remodeling [41], HSC mobilization to the circulation [37, 40, 42, 43], and HSC homing to the BM [44].

These collective findings delineate an attractive possibility of considering rMMP-9 as a radiomitigator for clinical applications involving SGS. In stark contrast to the immunogenic inflammatory response induced by entolimod that has limited its clinical development, MMP-9 is a natural endogenous human factor with no detected inflammatory activities and thus has clear advantages over entolimod as a radiomitigator. It is highly unlikely that the association of MMP-9 with tumor invasion and metastasis would have any relevance to its use as a radiomitigator since the pro-tumor effects are associated with chronic production [45] while the post-administration effects of MMP-9 as a radiomitigator would be considered shorter lasting.

**Fig. 6:**
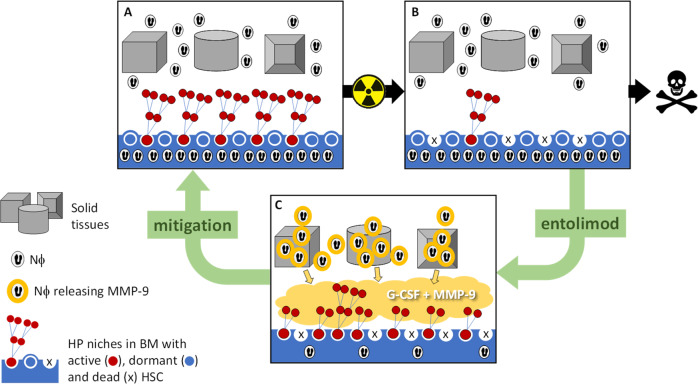
Proposed mechanism by which entolimod mitigates lethal ARS to hematopoiesis. Following lethal radiation damage, entolimod stimulates Nϕ to release MMP-9 which, in cooperation with G-CSF, accelerates the recovery of hematopoiesis.

The release of MMP-9 by Nϕ plays a pivotal role in TLR5 agonist-mediated regeneration of the HPS in response to radiation-induced damage (Fig. 6). However, MMP-9 is unlikely the sole driver of radiomitigation based on the high concentration of G-CSF and IL-6 in the circulation post-entolimod that stimulates HPS regeneration [14]. Based on prior work [21], MMP-9 likely augments the release of G-CSF to increase the number of HPPs in the BM. Currently, G-CSF is broadly used as an accelerator of hematopoietic restoration following myeloablative chemotherapy [46] and the G-CSF-based drugs Neupogen and Neulasta have been stockpiled as radiomitigators for emergency use [47]. Although IL-6 was shown to stimulate megakaryocytic precursor [48], its potent pyrogenic activity [49] has a controversial role as a likely mediator of flu-like syndrome in entolimod applications. Therefore, it would be important to test the efficacy of a combination of rMMP-9 and G-CSF as a potential new radiomitigator lacking the drawbacks and limitations of TLR5 agonists for mass casualty situations.

## Materials and methods

### Mice

Pathogen-free inbred BALB/c mice were obtained from The Jackson Laboratories or from our colony maintained at Roswell Park. BALB/c-*Tlr5*^*−/−*^ mice obtained from Dr. Shibata [24] and C57BL/6-GFP breeders [C57BL/6-Tg(UBC-GFP)30Scha/J] purchased from The Jackson Laboratories were all bred and maintained by Roswell Park Laboratory Animal Shared Resource (LASR). All mice were housed in microisolator cages in a laminar flow unit under ambient light. The Roswell Park Institutional Animal Care and Use Committee (IACUC) approved all procedures carried out in this study. Adult female mice between the ages of 11 and 12 weeks were assigned randomly to groups; group sizes were selected based on prior experience. No animals were excluded from further analysis in the reported studies.

### Generation of *Tlr5*^*−/−*^ bone marrow chimeric mice

BALB/c and *Tlr5*^*−/−*^ mice received a fractionated dose (3.75 Gy 3 h apart) of TBI using a 4000 Ci Cesium-137 source (J.L. Shepherd and Associates, San Fernando, CA) on a rotating platform to ensure even dose delivery to all tissues. Mice received syngeneic bone marrow transplantation by intravenous (i.v.) injection 24 h later. Mice rested for at least 9 weeks prior to enrollment into studies.

### Reagents

The TLR5 agonist entolimod (CBLB502) is a cGMP-manufactured drug product that was obtained from Cleveland BioLabs, Inc. [12]. We purchased mouse MethoCult GF and MethoCult without cytokines from STEMCELL Technologies, recombinant mouse G-CSF from Peprotech, rat IgG2a, κ (clone 2A3) and α-Ly-6G (clone 1A8) from BioXcell, Proteome Profiler Mouse XL Cytokine Array from R&D Systems, Duoset ELISA kits from R&D Systems, and recombinant mouse MMP-9 from BioLegend. All reagents were handled according to the manufacturer’s instructions.

### Radioprotection and radiomitigation studies

Mice received 7.5 Gy TBI similarly as described above for all studies except for non-survival radiomitigation studies in which mice received 5 Gy TBI. Mice received vehicle (PBS) or entolimod (1 μg) s.c. 30 min pre-TBI for radioprotection studies [12, 15, 19] or 24 h post-TBI for radiomitigation studies [13, 14].

### Nϕ depletion and isolation

To effectively deplete Nϕ, mice were intraperitoneally (i.p.) administered mAbs against Ly6G (100 μg/mouse) 18 h prior to vehicle or entolimod administration. Isotype-matched controls (rat IgG2a, κ) were given at the same dose and regimen as the depletion mAbs. Nϕ isolation and transfer were achieved by magnetic bead isolation from the bone marrow of naïve mice according to the manufacturer’s protocol using the ultrapure Ly6G microbead kit (Miltenyi Biotec). Purity was routinely greater than 98%. Nϕ were transferred via i.v. injection immediately before vehicle or entolimod administration.

### FACS staining and analysis

Single-cell suspensions of tissues were generated as outlined (Supplementary Table 1). Erythrocytes were lysed using a solution of ammonium chloride for 5 min at room temperature except for when Ter-119 was used in Panel 1. Live cells were counted by trypan blue exclusion, resuspended in phosphate azide buffer (PAB), and stained on ice for 10 min with TruStain fcX™ (clone 93, BioLegend for Panels 1 and 2) or rat serum (for Panel 3) followed by a cocktail of mAbs or isotype-matched controls for 15 min (Supplementary Table 2). We biotinylated entolimod with the EZ-Link™ Sulfo-NHS-LC-Biotinylation Kit (ThermoFisher). After staining, cells were washed once with PAB and fixed in 2% ultrapure formalin (Polysciences). Data was acquired within 48 h of fixation on a LSRII Fortessa instrument (Becton Dickinson), stored in Listmode format, and analyzed using WinList software (Verity House Software, Topsham ME).

### Enumeration of HPPs

Femurs and tibias were crushed with a sterilized mortar and pestle to generate a single cell suspension followed by erythrocyte lysis. Total live BM cells were counted by trypan blue exclusion and an equal number was plated in MethoCult GF (supports the growth of HPPs) or MethoCult with the addition of 50 ng/mL G-CSF (supports the growth of granulocyte/macrophage (G/M) progenitors [19]). The number of colonies were enumerated under a microscope on day 7 after plating and normalized to 50,000 total live BM cells for radiomitigation studies.

### Measurement of cytokine response

Serum was collected after centrifugation of naturally clotted whole blood for at least 1 h at room temperature. Serum and supernatants collected from ex vivo stimulated Nϕ were subjected to G-CSF, IL-6, and total MMP-9 ELISAs or R&D Systems Proteome Profiler Mouse XL Cytokine Array.

### Statistical evaluation

Experiments were not blinded and were repeated at least once. Data are presented as mean ± SEM of biological replicates. Differences between groups within experiments were analyzed in GraphPad Prism 9 software using unpaired Student’s *t*-test. Animal survival Kaplan–Meier curves were compared using the Log-rank test. A similar variance was observed between the groups that were statistically compared. Statistical significance between groups was defined as *P* < 0.05.

## Supplementary information

Signaling through TLR5 mitigates lethal radiation damage by neutrophil-dependent release of MMP-9

## Data Availability

The authors declare that all data supporting this study are available within the article and its Supplementary Information file, may be obtained from the corresponding author upon reasonable request, or in the case of our RNA-seq experiment comparing gene expression in mouse Nϕ or livers treated with vehicle or entolimod are available in NCBI’s Gene Expression Omnibus (GEO) database under accession number GSE179808 or GSE163748, respectively.
