# Supplementary material for: Signaling through TLR5 mitigates lethal radiation damage by neutrophil-dependent release of MMP-9
Source: Cell Death Discov. 2021 Sep 28;7:266. doi: 10.1038/s41420-021-00642-6 (PMC8478872; doi:10.1038/s41420-021-00642-6)
Supplement: Supplementary file 1 — Signaling through TLR5 mitigates lethal radiation damage by neutrophil-dependent release of MMP-9 [file 41420_2021_642_MOESM1_ESM.pdf]

Signaling through TLR5 mitigates lethal radiation damage by neutrophil-dependent release of MMP-9

Craig M. Brackett, Kellee F. Greene, Alyssa R. Aldrich, Nicholas H. Trageser, Srabani Pal, Ivan Molodtsov, Bojidar M. Kandar, Lyudmila G. Burdelya, Scott I. Abrams, and Andrei V. Gudkov

Supplementary Information

Material and Methods

Mice

Pathogen-free mice deficient for MyD88 (*Myd88*$^{-/-}$) on C57BL/6 background were a generous gift from Dr. Sandra Gollnick at Roswell. BALB/c-Tg (*IκBα-luc*)Xen reporter mice (aka NF-κB luciferase mice) similarly used by us [1] were originally purchased from Xenogen Corp. and bred in our colony at Roswell. Adult female mice between the ages of 11 and 12 weeks were assigned randomly to groups; group sizes were selected based on prior experience. No animals were excluded from further analysis in the reported studies.

Cell Lines

The authenticated mouse endothelial cell line 2H-11 was purchased from the ATCC and cultured according to their recommendations under conditions free of mycoplasma contamination.

Reagents

We purchased Hemavet compatible kits for complete blood count (CBC) from Drew Scientific, α-L-selectin (clone MEL-14) and its isotype control (rat IgG2a,κ) from BioXcell, α-ICAM-1 (clone 3E2) and its isotype control (Armenian hamster IgG1, κ) from BD Biosciences, Duoset ELISA kits from R&D Systems, Vybrant CFSA SE cell tracer kit from ThermoFisher, and Bright-Glo luciferase assay system from Promega. All reagents were handled according to the manufacturer's instructions.

FACS staining and analysis

For *ex vivo* trafficking studies, Nϕ that were isolated and treated were then stained with a mixture of FITC L-selectin (MEL-14) and PE-Cy7 CD11b (M1/70) antibodies, both of which were purchased from BioLegend. For *in vivo* trafficking studies, single cell suspension of total liver cells was stained with Panel 2 (Nϕ response) antibodies: APC-efluor780 CD45, PE-Cy7 CD11b, PerCPCy5.5 Ly-6C, and Ax700 Ly-6G.

Nϕ trafficking studies

For L-selectin and ICAM-1-mediated Nϕ recruitment to the liver post-entolimod, cohorts of intact mice were given blocking antibodies to L-selectin (100 μg via intraperitoneal injection) or ICAM-1 (50 μg via intravenous injection) or appropriate isotype controls 30 minutes prior to entolimod. Livers were collected from mice 2h post-treatment with entolimod or vehicle to enumerate the number of Nϕ by FACS. For entolimod-stimulated Nϕ binding to 2H-11 endothelial cells, Nϕ were isolated from the BM of intact mice and stained with CFDA dye prior to entolimod treatment and incubation with 2H-11 cells. Intensity of CFDA was measured on a Perkin Elmer Victor plate reader as a surrogate for Nϕ binding to 2H-11 cells.

RNA-seq-based comparative transcriptomic in mouse Nϕ and livers

Following magnetic isolation from BM, Nϕ were treated with vehicle or entolimod (1 ng/mL) for 30 minutes and then washed once with PBS and flash frozen. Livers were collected at 30 minutes after treatment of mice with vehicle or entolimod (0.3 μg) and flash frozen. RNA was extracted from Nϕ and livers and RNA-seq was performed similarly as described by us [2]. Briefly, the quality of the sequencing data was assessed via FastQC (http://www.bioinformatics.babraham.ac.uk/projects/fastqc). Reads were aligned to the mouse reference genome (UCSC mm10/GRCm38) with STAR RNA-seq aligner [3] using annotation from the same source. Reads were counted using featureCounts [4] using the same annotation. Differential gene expression and normalized counts were calculated using DESeq2 [5]. Further DE comparison, analysis, and visualization were performed using Python programming language.

Supplementary Tables

Supplementary Table 1. Enzyme solutions and methods to digest tissues for FACS

Tissue(s)	Enzyme Solution	Method
Bladder and kidneys	1 mg/mL collagenase IV and 0.02 mg/mL DNase, 10 mL per tissue	Shaking for 1 hour at 37℃
Lungs	1 mg/mL collagenase IV and 0.02 mg/mL DNase, 7 mL per tissue	
Pancreas	0.1 mg/mL collagenase IV, 10 mL per tissue	
Heart	20 mM $CaCl_2$, 2 mg/mL collagenase IV, 1.2 U/mL Dispase II, 10 mL per tissue	
Liver	50 µM EGTA, 0.2 mg/mL collagenase IV	Perfusion followed by shaking for 30 minutes at 37℃
BM	N/A	Crushed with sterilized mortar and pestle
Spleens	N/A	Mechanical disruption
Blood	N/A	10% heparin

Supplementary Table 2. Antibody Panels for FACS

Panel	Antibodies	Vendor	Catalog Number
#1 (TLR5 expression)	PerCPCy5.5 CD45 (clone 30-F11)	BioLegend	103132
	Brilliant Violet (BV) 605 CD11c (clone N418)	BioLegend	117334
	APC-Cy7 Ly-6G (clone 1A8)	BioLegend	127624
	PE-Cy7 CD11b (clone M1/70)	BioLegend	101216
	APC F4/80 (clone BM8)	BioLegend	123116
	Alexa Fluor (Ax) 700 B220 (clone RA3-6B2)	BioLegend	103232
	APC Ter-119 (clone Ter-119)	ThermoFisher	17-5921-82
	PE-Cy7 CD41 (clone MWReg30)	BioLegend	133916
	Streptavidin PE	ThermoFisher	12-4317-87
#2 (Neutrophil response)	APC-efluor780 CD45 (clone 30-F11)	ThermoFisher	47-0451-82
	BV421 CD11b (clone M1/70)	BD Biosciences	562605
	Ax700 Ly-6G (clone 1A8)	BioLegend	127622
	PerCPCy5.5 Ly-6C (clone HK1.4)	BioLegend	128012
#3 (HSCs)	biotin lineage panel (clones Ter-119, M1/70, RB6-8C5, 145-2C11, RA3-6B2)	BioLegend	133307
	Streptavidin PE-Cy7	BD Biosciences	557598
	APC CD135 (clone A2F10)	BioLegend	135310
	BUV395 CD117 (clone 2B8)	BD Biosciences	564011
	PE Ly6A/E (clone D7)	BD Biosciences	553108
	BV785 CD127 (clone A7R34)	BioLegend	135037
	BV421 CD34 (clone SA376A4)	BioLegend	152208
	BV510 CD16/32 (clone 93)	BioLegend	101333
#4 (Neutrophil differentiation)	biotin lineage panel (clones Ter-110, 145-2C11, RA3-6B2, Sca-1)	BioLegend	133307
	Streptavidin PE	ThermoFisher	12-4317-87
	Ax647 CD115 (clone AFS98)	BioLegend	135530
	PerCPCy5.5 Gr-1 (clone RB6-8C5)	BioLegend	108428
	PE-Cy7 CD11b (clone M1/70)	BioLegend	101216
	BV510 CXCR4 (clone 2B11/CXCR4)	BD Biosciences	563468
	BV421 CXCR2 (clone V48-2310)	BD Biosciences	566622
	Ax700 Ly-6G (clone 1A8)	BioLegend	127622

Supplementary Figures

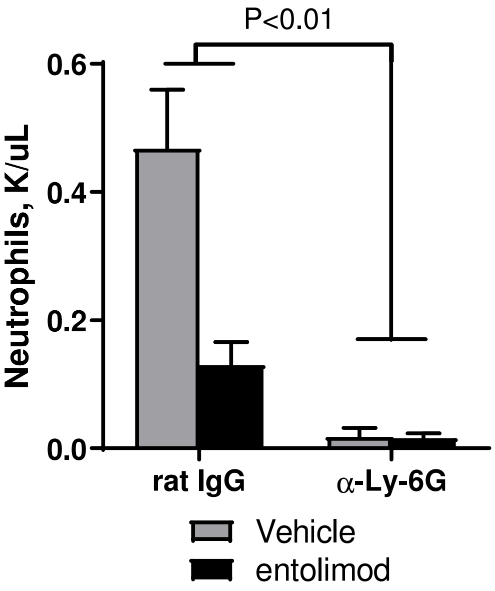

Supplementary Figure S1. Effective depletion of Nϕ with α-Ly-6G antibody. The experimental design outlined in Figure 1E was used. Nϕ were measured in blood of mice given rat IgG or α-Ly-6G by complete blood cell count with differential 2h post-treatment with vehicle or entolimod. Error bars represent mean ± SEM; P-values were determined by Student's *t* test; n = 5 mice /group.

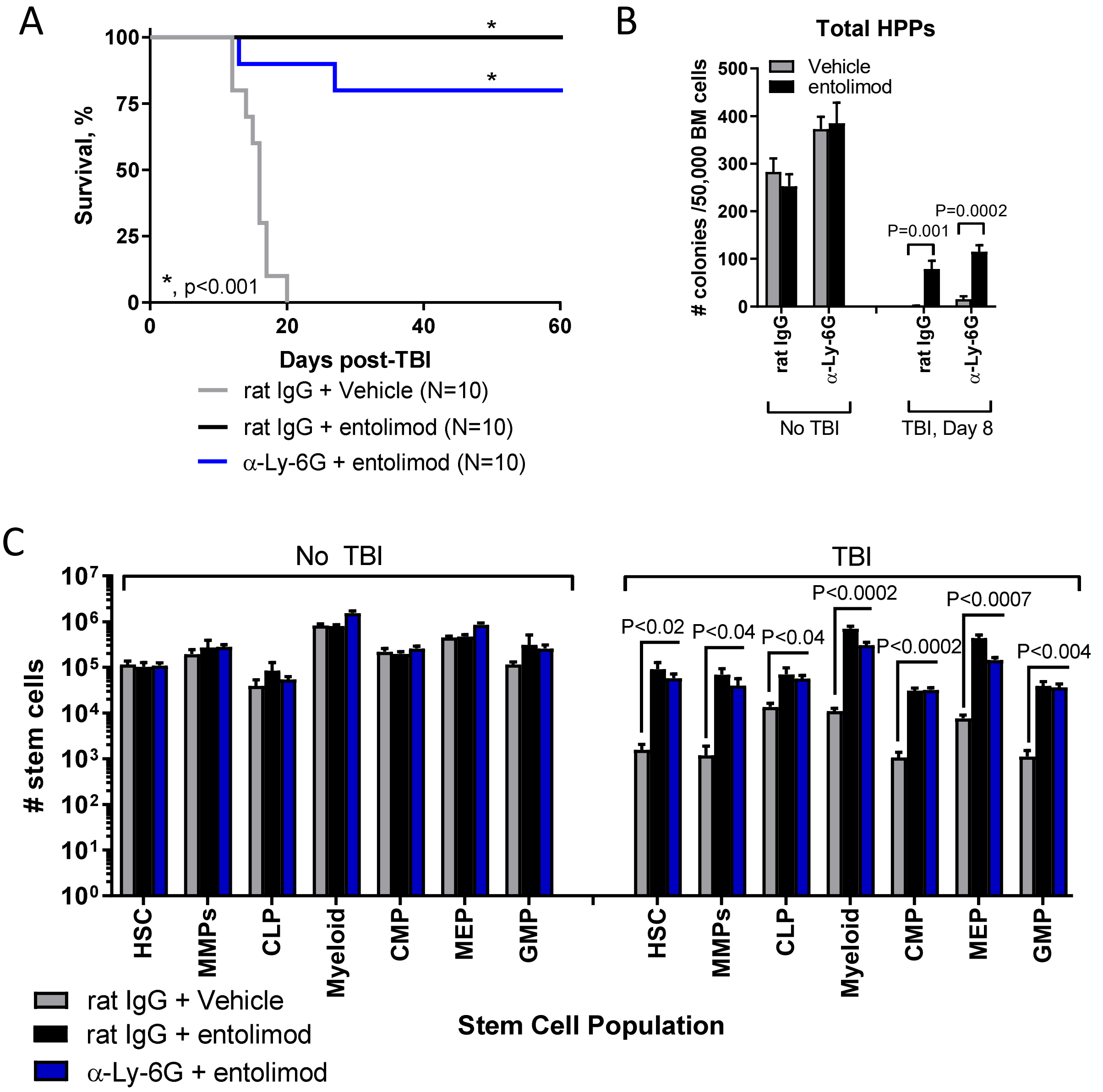

Supplementary Figure S2. Nϕ do not mediate the radioprotective activities of entolimod in outbred NIH-S mice. The experimental design outlined in Figure 1A was used. (A) Survival by Kaplan-Meier curves in mice treated with entolimod in the presence (rat IgG) or absence (α-Ly-6G) of Nϕ. P-values were determined by Log-rank test; *P<0.001. (B) Measurement of total HPPs by MethoCult (n = 4-5 mice /group) and (C) absolute number of stem cell populations by flow cytometry in the presence (rat IgG) or absence (α-Ly-6G) of Nϕ on day 8 post-treatment with vehicle or entolimod (n = 4-5 mice /group). Error bars represent mean ± SEM; P-values were determined by Student's *t* test.

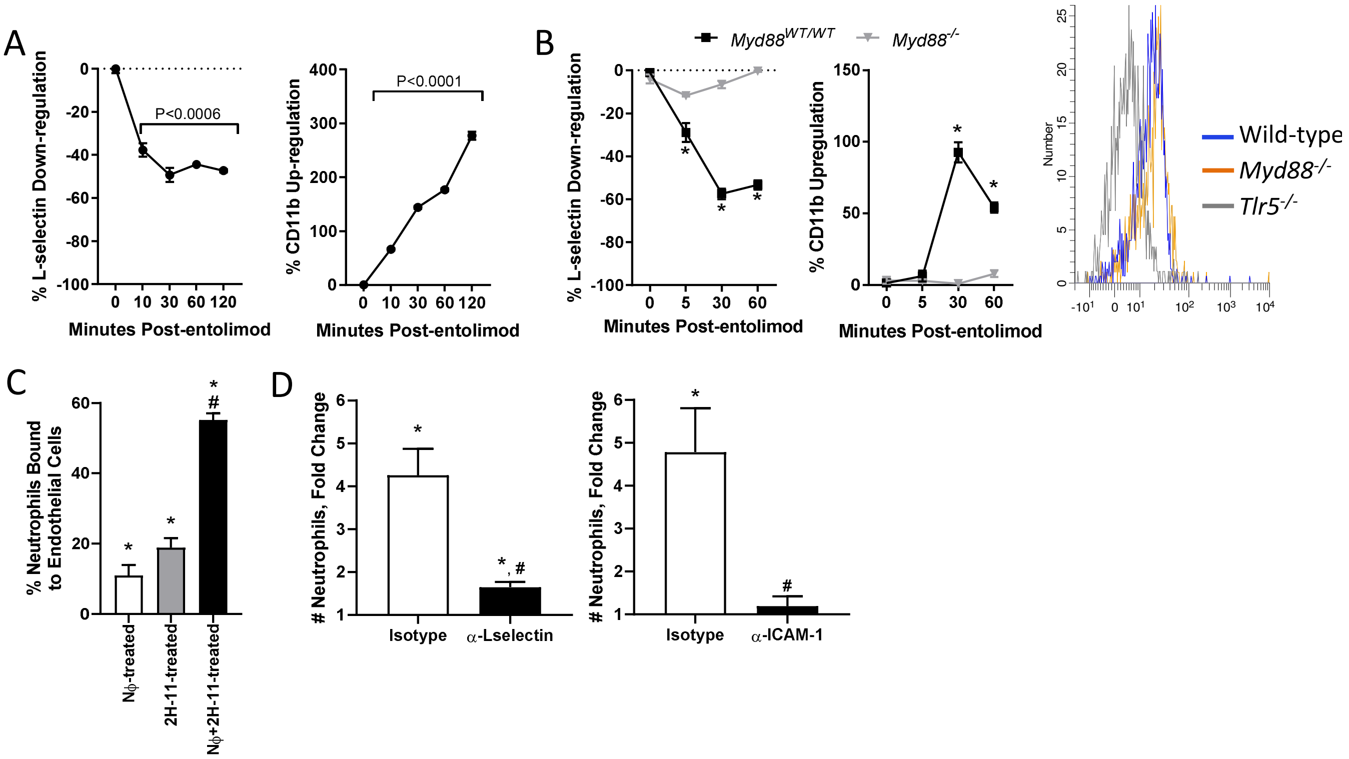

Supplementary Figure S3. Entolimod increases the trafficking properties of Nϕ. (A) Nϕ magnetically isolated from the bone marrow of *Tlr5*$^{WT/WT}$ mice were treated *ex vivo* with vehicle or entolimod (8 pM) for the indicated time points and stained for flow cytometry with mAb against L-selectin and CD11b. Percent L-selectin down-regulation was calculated using the formula [($MFI_{entolimod}$ / $MFI_{vehicle}$)-1] x100%. Percent CD11b up-regulation was calculated using the formula ($MFI_{entolimod}$ / $MFI_{vehicle}$) x100%; n = 3 mice /group. (B) Nϕ magnetically isolated from the bone marrow of *Myd88*$^{WT/WT}$ and *Myd88*$^{-/-}$ mice were treated *ex vivo* with vehicle or entolimod (8 pM) for the indicated time points and stained for flow cytometry with mAb against L-selectin and CD11b. L-selectin down-regulation and CD11b up-regulation were calculated as in (A); n = 3 mice /group. Blood from *Myd88*$^{WT/WT}$, *Myd88*$^{-/-}$, and *Tlr5*$^{-/-}$ mice was RBC lysed and stained with our b-entolimod flow cytometry "sandwich" platform to measure TLR5 expression on Nϕ ($CD45^{+}$ $CD11b^{+}$ Ly-$6C^{lo}$ Ly-$6G^{hi}$). (C) Nϕ isolated from the bone marrow of *Tlr5*$^{WT/WT}$ mice were fluorescently labeled with 10 μM CFDA. Endothelial cells (2H-11) were either left untreated or treated for 30 minutes with vehicle or entolimod (3 pM), washed once, and incubated for another 4.5h prior to the addition of CFDA-labeled Nϕ. Fluorescence was measured after washing four times. Percent Nϕ binding was calculated using the formula ($Fluorescence_{entolimod}$ / $Fluorescence_{vehicle}$) x 100% after which background fluorescence was subtracted from vehicle and entolimod treated fluorescence values; n = 3 mice /group. (D) L-selectin and CD11b mediated recruitment of Nϕ ($CD45^{+}$ $CD11b^{+}$ Ly-$6C^{+}$ Ly-$6G^{hi}$) to the liver 2h post-entolimod. Cohorts of mice were given blocking antibodies to L-selectin or ICAM-1 or appropriate isotype controls 30 minutes prior to entolimod. Fold change was calculated based on the absolute number of Nϕ in mice treated with entolimod versus vehicle for each antibody treatment group; n = 4-8 mice /group. Error bars represent mean ± SEM and p-values were determined by Student's *t* test.

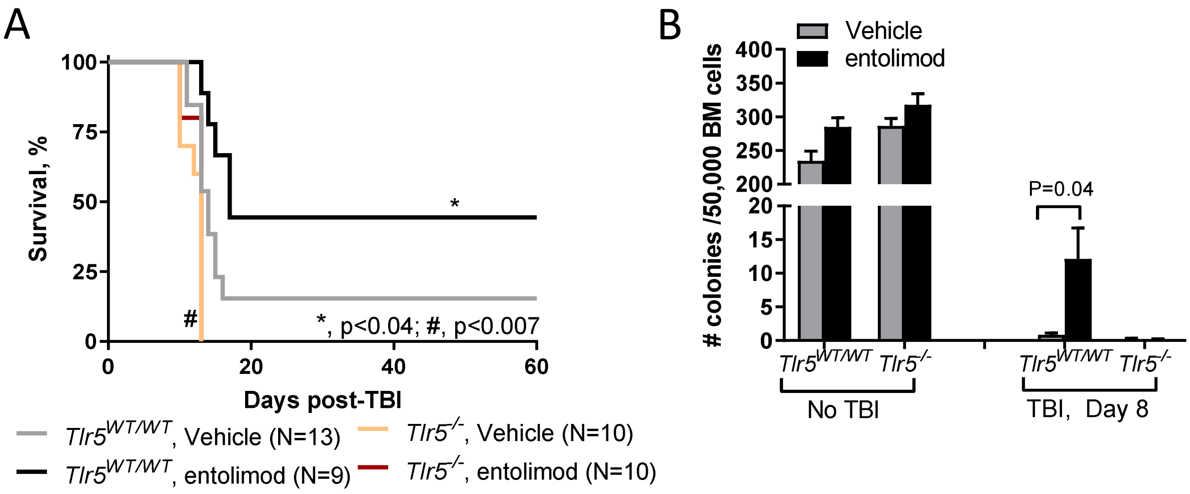

Supplementary Figure S4. The radiomitigative activity of entolimod is TLR5-dependent. (A) Survival by Kaplan-Meier curve in *Tlr5*$^{WT/WT}$ and *Tlr5*$^{-/-}$ mice treated with vehicle or entolimod 24h post-TBI. P-values were determined by Log-rank test; *P<0.04 when comparing *Tlr5*$^{WT/WT}$ and #P<0.007 when comparing *Tlr5*$^{WT/WT}$ to *Tlr5*$^{-/-}$. (B) Measurement of total HPPs by MethoCult from the BM of *Tlr5*$^{WT/WT}$ and *Tlr5*$^{-/-}$ mice on day 7 post-treatment. Cohorts of mice given TBI were treated with vehicle or entolimod 24h later and in the case of the no TBI cohort mice were treated in a similar manner (n = 6-10 mice /group). Error bars represent mean ± SEM; P-values were determined by Student's *t* test.

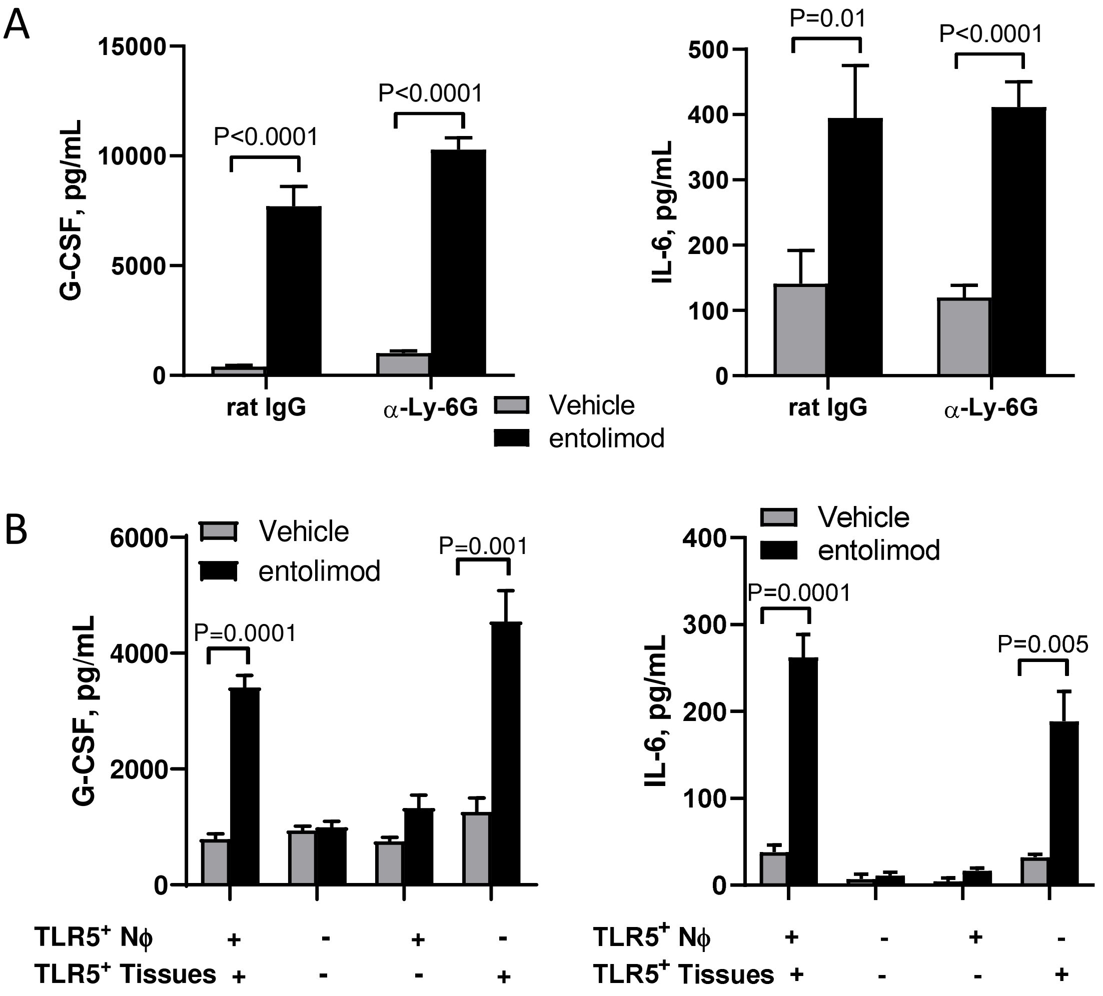

Supplementary Figure S5. TLR5 stimulation on non-hematopoietic cells releases G-CSF and IL-6. Measurement of serum levels of G-CSF and IL-6 by ELISA in (A) the presence (rat IgG) and absence (α-Ly-6G) of Nϕ (n = 10 mice /group) or (B) *Tlr5*$^{-/-}$ bone marrow chimeric mice 2h post-treatment with vehicle or entolimod (n = 4-6 mice /group). Mice received TBI 24h prior to vehicle or entolimod treatment. Error bars represent mean ± SEM; P-values were determined by Student's *t* test.

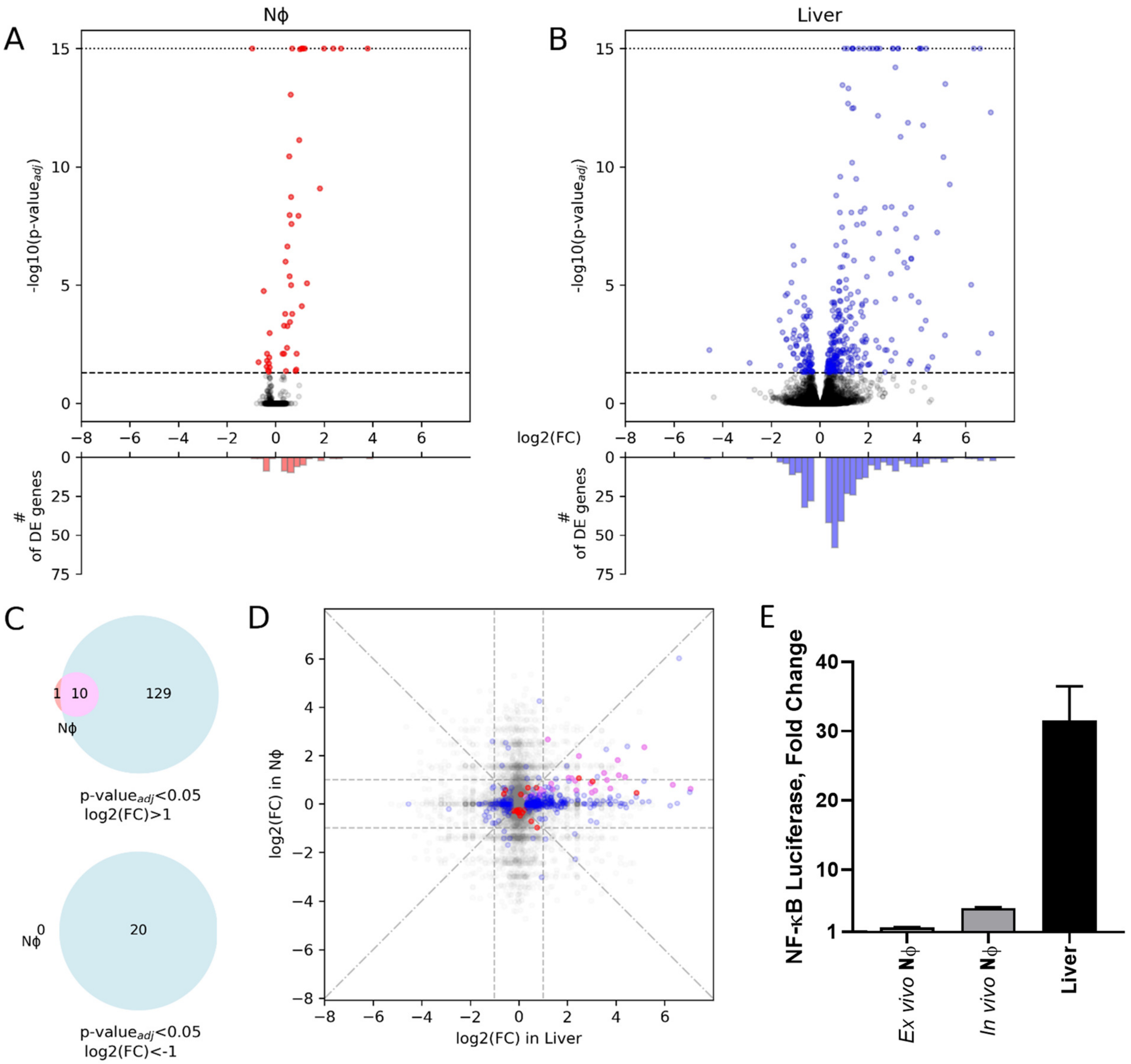

Supplementary Figure S6. Entolimod causes minimal transcriptional changes in Nϕ. Volcano plots showing differentially expressed genes in (A) Nϕ (n = 2 mice /group) and (B) liver (n = 3 mice /group) with colored dots showing genes with statistically significant differential expression (red for Nϕ, blue for liver). Dashed line show p-value$_{adj}$<0.05 significance threshold; for visualization purposes, all p-values$_{adj}$ below 1e-15 were assigned this level (shown as dotted line). Below the volcano plots are histograms of distribution of log2(FC) in statistically significant differential expressed genes in Nϕ and liver. (C) Venn diagrams for list of genes which are up-regulated (p-value$_{adj}$<0.05, log2(FC)>1; top) or down-regulated (p-value$_{adj}$<0.05, log2(FC)<-1; bottom) in Nϕ (red) and liver (blue) and violet used for intersection. (D) Scatter plot for per-gene log2(FC) in Nϕ and liver. Blue dots show genes which are DE with p-value$_{adj}$<0.05 in Nϕ only, red – in liver only, and violet – genes which are DE with p-value$_{adj}$<0.05 both in Nϕ and liver. Dashed lines show log2(FC) = -1/1 thresholds; dash-and-dotted lines show thresholds of equal absolute log2(FC) values. (E) Fold change in NF-κB driven luciferase activity in Nϕ (n = 4-6 mice /group) and liver (n = 5 mice) post-entolimod. *Ex vivo* indicates that Nϕ were treated with vehicle or entolimod for 3h after isolation prior to luciferase measurement. I*n vivo* indicates that mice were treated with vehicle or entolimod for 3h followed by Nϕ isolation and immediate luciferase measurement. For liver, luciferase activity was measured in protein lysates collected from mice 3h post-treatment with vehicle or entolimod. Error bars represent mean ± SEM.

Supplementary References

1. Burdelya LG, Brackett CM, Kojouharov B, Gitlin, II, Leonova KI, Gleiberman AS, et al. Central role of liver in anticancer and radioprotective activities of Toll-like receptor 5 agonist. Proceedings of the National Academy of Sciences of the United States of America. 2013;110:E1857-66.
2. Mett V, O VK, I AB, Molodtsov I, C MB, L GB, et al. A deimmunized and pharmacologically optimized Toll-like receptor 5 agonist for therapeutic applications. Commun Biol. 2021;4:466.
3. Dobin A, Davis CA, Schlesinger F, Drenkow J, Zaleski C, Jha S, et al. STAR: ultrafast universal RNA-seq aligner. Bioinformatics. 2013;29:15-21.
4. Liao Y, Smyth GK, Shi W. featureCounts: an efficient general purpose program for assigning sequence reads to genomic features. Bioinformatics. 2014;30:923-30.
5. Love MI, Huber W, Anders S. Moderated estimation of fold change and dispersion for RNA-seq data with DESeq2. Genome Biol. 2014;15:550.
